# Effectiveness of a structured special quality management program on insulin injection practices among community-dwelling patients with diabetes in a county district: a pre-post intervention study

**DOI:** 10.3389/fpubh.2026.1809596

**Published:** 2026-04-29

**Authors:** Xuehang Lou, Xudan Wu, Sha Jia

**Affiliations:** Department of Internal Medicine, Dongyang Maternal and Child Health Hospital, Dongyang, Zhejiang, China

**Keywords:** community, diabetes, insulin injection, questionnaire survey, special management

## Abstract

**Objective:**

To investigate the current status of insulin injection among patients with diabetes in a county-level community through questionnaires and to implement a targeted special quality management (QM) program based on the findings.

**Methods:**

Using a one-group pre-post study design, 297 patients receiving insulin injections were randomly selected from the local community’s chronic disease management database. A baseline insulin injection-related questionnaire survey was conducted. A multidisciplinary collaborative management team (MCMT) was established to analyze the results and implement a structured special QM program. The team developed an insulin injection quality evaluation system and carried out interventions including systematic health education, standardized operation training, and regular quality supervision with feedback. After a one-year QM period, a second questionnaire survey was administered. To assess the sustainability of the intervention effects, a follow-up evaluation was conducted 6 months after the completion of the QM program (i.e., at 18 months from baseline), using the same questionnaire and HbA1c measurement. These data will be reported in a subsequent analysis to determine whether improvements in knowledge, injection practices, and glycemic control were maintained over time. Baseline data were also indirectly compared with recent international Insulin Injection Technique Questionnaire (ITQ) data for contextualization.

**Results:**

After the QM intervention, significant improvements were observed in knowledge and operational levels concerning diabetes, insulin, insulin storage, and insulin injection (*p* < 0.05). Glycated hemoglobin (HbA1c) levels decreased from 7.13% (SD = 1.74%) at baseline to 5.93% (SD = 1.00%) post-intervention, with a mean reduction of 1.20% (95% CI, 0.98–1.42%, *t* = 10.54, *p* < 0.001), indicating a statistically and clinically significant improvement.

**Conclusion:**

Addressing insulin injection problems identified in the county community through establishing a targeted special QM program is beneficial for improving diabetes control and management at the community level.

## 1 Introduction

Diabetes is a chronic progressive disease leading to various complications such as stroke, heart failure, and uremia, severely impacting patients’ physical and mental health, reducing quality of life, and increasing societal and familial burdens. The prevalence of adult diabetes in China increased from 10.9% in 2013 to 12.4% in 2018 (1). Research identifies diabetes as a central disease within comorbid chronic condition clusters in communities (2), significantly elevating risks of premature death, hospitalization, disability, polypharmacy, and worsened quality of life (3). While clinical care focuses on treatment, long-term effective management of chronic diseases increasingly relies on community public health systems, presenting challenges for community chronic disease management.

Insulin injection plays a crucial role in glycemic control, substantially delaying diabetes progression and complication onset. However, global data from the Insulin Injection Technique Questionnaire (ITQ) indicate that suboptimal injection practices-such as needle reuse, incorrect site rotation, and improper storage-are widespread, affecting glycemic control and increasing the risk of complications (4, 5). In China, these issues are particularly pronounced in community and primary care settings due to limited access to specialized education and training (6). Previous interventions have largely focused on hospital-based populations, with few addressing community-dwelling patients through structured, multidisciplinary quality management programs (7). Theoretical frameworks such as the Plan-Do-Study-Act (PDSA) cycle and the Chronic Care Model have been successfully applied in diabetes management to support continuous quality improvement and patient engagement (8, 9).

For patients on insulin therapy, clinicians and community physicians often prioritize dosage adjustment, type selection, and timing to achieve glycemic targets, potentially overlooking potential issues throughout the entire injection process-from pen preparation to post-injection handling. Incorrect insulin injection practices and storage can lead to adverse events such as lipohypertrophy, hypoglycemia, and poor glycemic control (10).

This study aimed to use questionnaires to analyze current problems in insulin injection practices at the primary community level, conduct targeted data analysis, and implement a special QM program for continuous quality improvement to enhance injection standardization and evaluate its effectiveness in a real-world community setting.

## 2 Materials and methods

### 2.1 Patient data

From December 2022 to March 2023, patients with diabetes receiving insulin injections, registered in the county’s public health service chronic disease management system, were randomly screened using a computer-generated random number sequence. The source population consisted of 1,248 registered patients with diabetes who were on insulin therapy across 12 community health service centers in the county. A total of 297 valid questionnaire responses were obtained, representing a response rate of 89.7% (297/331 approached). Participants were representative of the broader community population in terms of age, sex, and diabetes duration based on available registry data.

### 2.2 Inclusion and exclusion criteria

Inclusion criteria: (1) Diagnosis meeting the Chinese Diabetes Prevention and Treatment Guidelines criteria; (2) Requirement for long-term insulin injection therapy; (3) Permanent residency in the community; (4) Voluntary participation with signed informed consent. Exclusion criteria: (1) Presence of acute complications; (2) Cognitive impairment; (3) Requiring only short-term insulin injection; (4) Use of insulin pumps.

### 2.3 Research methods

#### 2.3.1 Establishment of a community diabetes insulin injection special QM team

A QM team was established comprising clinical physicians, endocrine nurses, and community responsible physicians. This multidisciplinary collaboration aimed to conduct the survey and QM work. The involvement of community physicians, with their established rapport in chronic disease management, enhanced patient trust and compliance. All team members had over 10 years of work experience.

#### 2.3.2 Questionnaire survey

The questionnaire was self-designed by the research team based on the Chinese Guidelines for Diabetes Drug Injection Technology (2016 Edition) and relevant literature. Content validity was evaluated by three endocrinology experts, yielding a Content Validity Index (CVI) of 0.92. A pilot test with 30 patients showed a Cronbach’s *α* coefficient of 0.835, indicating good reliability. Exploratory factor analysis extracted three common factors (knowledge, operation, storage), with a cumulative variance contribution rate of 71.5%, indicating good structural validity. (The full English version of the questionnaire is provided as Supplementary File 1).

After training, QM team members established contact via WeChat Work with enrolled patients or their families. Questionnaires were administered “one-to-one, face-to-face” using online links. Standardized instructions were provided, explaining the study’s purpose. Patients were instructed to answer based on their actual injection practices. Questionnaires were completed independently by patients or accompanying family members. Collected data were retrieved, analyzed, and checked for logic. After the initial survey and data analysis, the QM intervention was implemented. A second questionnaire was administered after 1 year of QM.

Questionnaire content: (1) Demographics: Gender, age, education level, diagnosis, diabetes duration, insulin injection duration. (2) Insulin Injection Status Survey: (a) Knowledge: Received insulin education? Received educational materials? Perception of insulin as a dangerous drug? Awareness of consequences of improper injection? (b) Operation: Received nurse-led injection training? Correct pen assembly? Appropriate site selection? Site disinfection? Single-use of needles? Regular injection assessment? Proper disposal of used pen needles? (c) Home Storage: Maintain home insulin stock? Spare insulin refrigerated? Opened insulin stored in fixed location? Used within stipulated period after opening?

#### 2.3.3 Implementation of the community diabetes insulin injection special QM program

**Figure 1:**
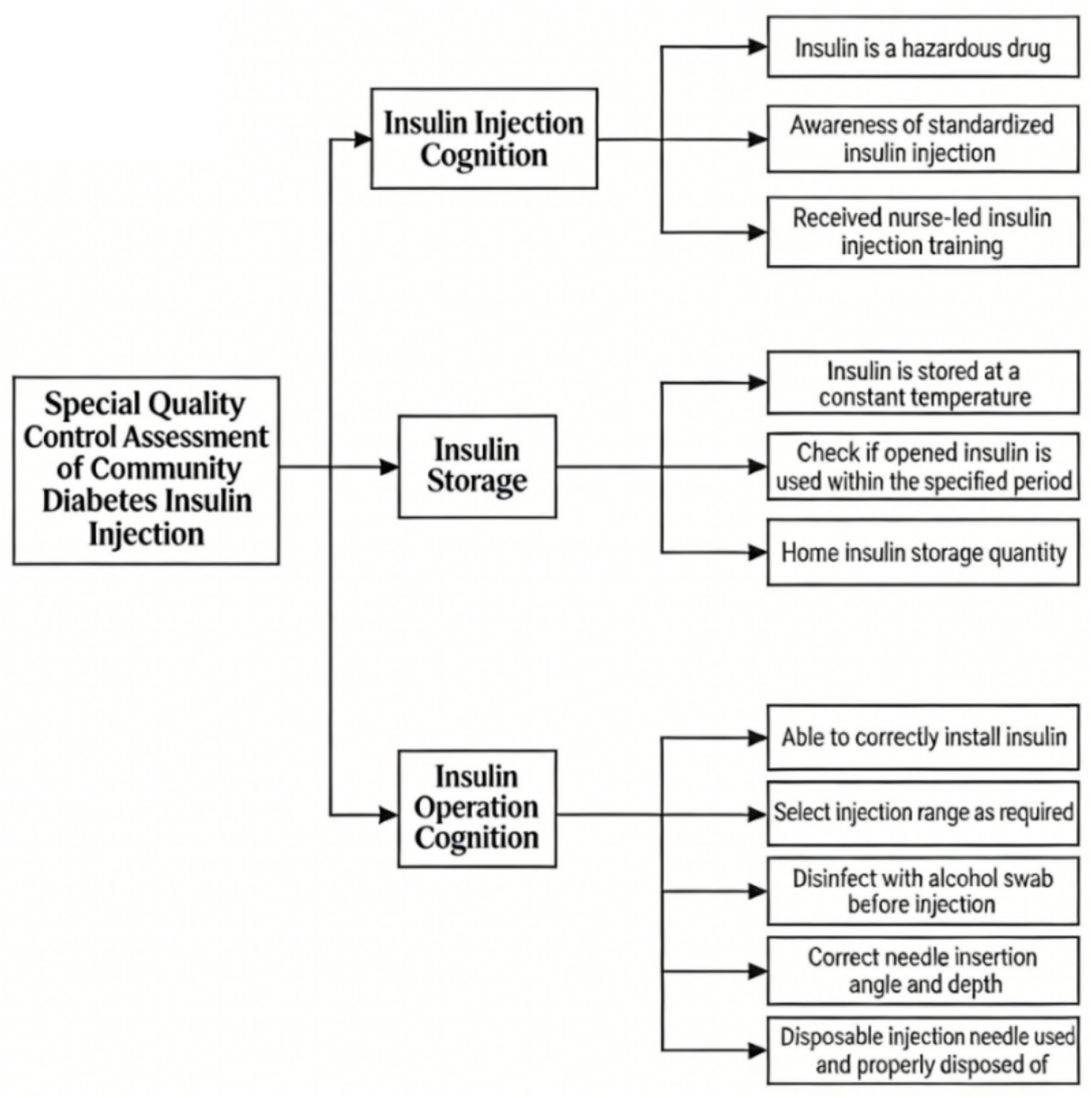
Quality evaluation form for insulin injection management in community diabetes care. This form was used by the multidisciplinary team during home visits and outpatient checks to systematically assess and document patients’ insulin injection knowledge, storage practices, and operational skills.

Based on survey results and literature review, the MCMT developed a quality evaluation form (Figure 1) with 3 first-level indicators (Knowledge Assessment, Insulin Storage Assessment, Insulin Injection Operation Assessment) and 11 s-level indicators.

Based on baseline findings, the MCMT designed and implemented a structured, multi-module intervention (Table 1). The 12-month intervention included monthly group education, quarterly one-on-one operation training, and bi-annual quality supervision and feedback sessions. All training was conducted by uniformly trained endocrine specialist nurses and community physicians using standardized materials and checklists. To ensure the fidelity and replicability of the intervention, all training sessions followed a pre-defined curriculum. The content for health education and operational training was standardized in an intervention manual, which included slide decks, pamphlets, and instructional videos. Education and training content focused on areas with low knowledge rates or non-standard practices identified in the survey. Basic knowledge was reinforced through individualized guidance.

**Table 1:** Structured special quality management intervention plan.

Intervention module	Specific content	Implementation methods	Frequency	Responsible personnel
Health education	Insulin pharmacology, storage requirements, injection risks, complication prevention	Group lectures, pamphlets, WeChat push notifications	Monthly	Clinical doctors, community physicians
Operation training	Pen assembly, site rotation, injection angle/depth, needle handling	One-on-one demonstration, video teaching, return demonstration	Quarterly	Endocrine specialist nurses
Quality supervision	Injection technique assessment, storage condition check, questionnaire review	Home visits, outpatient checks, online supervision	Every 6 months	All MCMT members
Feedback mechanism	Personalized problem analysis, success case sharing, continuous improvement plans	Group discussions, case analysis meetings	Quarterly	MCMT coordinator

#### 2.3.4 Sample size calculation

The required sample size was calculated using the formula for paired proportions in a pre-post intervention design. This calculation was based on data from a pilot study involving 30 patients, which showed that the proportion of patients demonstrating correct insulin injection technique was 45% at baseline. The intervention was anticipated to increase this proportion to 65% post-intervention. To detect this absolute difference of 20% with a two-sided significance level (α) of 0.05 and a statistical power (1-β) of 0.90, a minimum of 260 participants was required. To account for a potential 15% dropout rate during the 1-year follow-up, the target enrollment was adjusted to 300 participants. The final study sample of 297 participants who completed both assessments exceeded the minimum required sample size, ensuring adequate power for the primary analyses. For the comparison of paired pre-post intervention data, statistical significance was evaluated using the McNemar test for categorical variables (e.g., questionnaire items) and the paired *t*-test for continuous variables (e.g., HbA1c). The effect size for the primary outcome of correct injection technique was calculated using Cohen’s d (or an appropriate measure for proportions). For the change in HbA1c, the mean difference and its 95% confidence interval are reported in the results section to provide a measure of the magnitude and precision of the intervention effect.

#### 2.3.5 Study flow and participant progression

**Figure 2:**
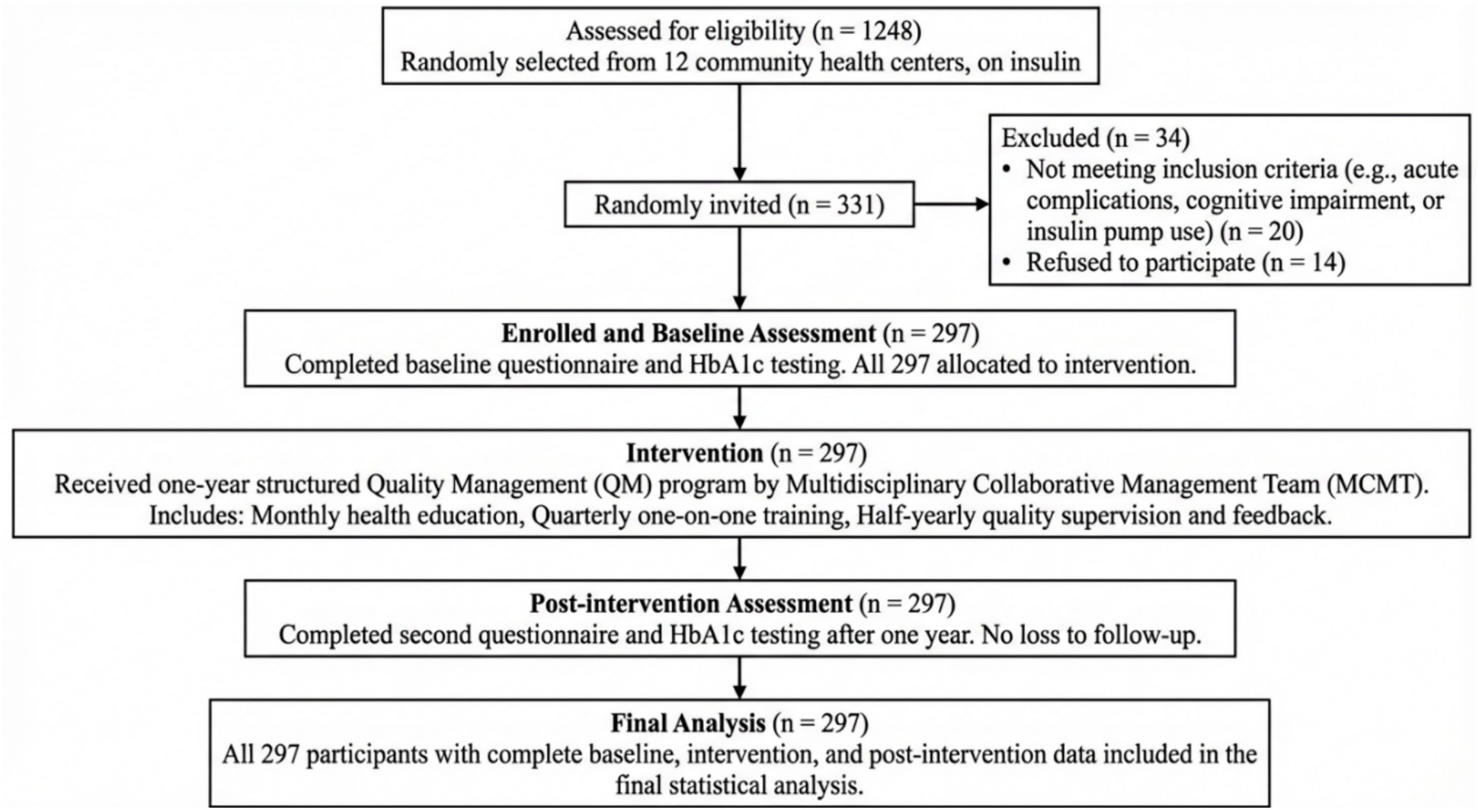
Flow diagram of participant recruitment, intervention, and assessment in the pre-post study. This flowchart summarizes participant progression. From a source population of 1,248 eligible patients, 331 were randomly approached, of whom 297 were enrolled after excluding 34 (20 ineligible, 14 declined). All 297 participants completed the baseline assessment, received the full one-year QM intervention, and completed the 12-month follow-up assessment, resulting in 297 participants included in the final analysis.

To enhance the clarity of the research process, a flow diagram has been added to illustrate participant progression through the study. The diagram details the stages of recruitment, baseline assessment, the one-year quality management intervention, and the post-intervention assessment, including the number of participants at each stage. This flow diagram is presented as Figure 2.

#### 2.3.6 HbA1c measurement

Venous blood samples were collected from all participants at baseline and at the 12-month post-intervention follow-up. HbA1c levels were analyzed at the central laboratory of Dongyang Maternal and Child Health Hospital using high-performance liquid chromatography (HPLC) with a Bio-Rad D-10™ Hemoglobin Testing System, which is certified by the National Glycohemoglobin Standardization Program (NGSP). To ensure the validity of the HbA1c results, all samples were processed within 24 h of collection, and the laboratory participated in external quality assurance programs. The intra-assay and inter-assay coefficients of variation were < 3%, confirming high analytical precision.

### 2.4 Statistical analysis

All statistical analyses were performed using SPSS version 27.0 (IBM Corp., Armonk, NY, United States). For the primary analysis comparing pre- and post-intervention outcomes, the paired *t*-test was used for normally distributed continuous data, such as HbA1c. The McNemar test, which is specifically designed for paired nominal data, was used to compare changes in categorical questionnaire responses (e.g., correct/incorrect knowledge or practice) from baseline to follow-up. A *p*-value of less than 0.05 was considered statistically significant for all tests.

### 2.5 Study design limitations and mitigation

This study used a one-group pre-post design without a concurrent control group. To partially address this, baseline data were indirectly compared with 2022 ITQ global data to contextualize local issues. A cluster randomized controlled trial is planned for future research.

## 3 Results

### 3.1 Basic characteristics of survey participants

Among the 297 participants, 171 (57.58%) were male and 126 (42.42%) female. Age distribution: 192 (64.65%) were over 61 years old. Education level: 190 (63.97%) had junior high school education or below, 107 (36.03%) had high school or above. Mean diabetes duration at baseline was 9.00 ± 5.65 years, and mean insulin injection duration was 4.49 ± 3.03 years. Disease duration was positively correlated with insulin injection duration (Spearman’s Rho = 0.56, *p* < 0.001). Details are shown in Table 2.

**Table 2:** Basic characteristics of questionnaire participants [*n* = 297 (percentage, %)].

Item	Group	Number (%)	Item	Group	Number (%)
Gender	Male	171 (57.58)	Age	≤50 years	28 (9.43)
	Female	126 (42.42)		51–60 years	77 (25.93)
Education	Primary or below	74 (24.92)		61–70 years	83 (27.95)
	Junior high	116 (39.06)		71–80 years	76 (25.59)
	Senior high	70 (23.57)		≥81 years	33 (11.11)
	College	5 (1.68)	Diagnosis	Type 1 diabetes	12 (4.04)
	Bachelor or above	32 (10.77)		Type 2 diabetes	285 (95.96)

### 3.2 Questionnaire results

#### 3.2.1 Seven items showed good baseline knowledge/performance

#### 3.2.2 Comparison of questionnaire results before and after the special QM intervention

All 297 patients who completed the initial survey participated in the QM program without dropouts and completed the second questionnaire.

##### 3.2.2.1 Comparison of knowledge assessment before and after QM

Insulin-related knowledge improved significantly post-intervention (*p* < 0.05).

##### 3.2.2.2 Comparison of insulin injection operation assessment before and after QM

Most operational items showed significant improvement (*p* < 0.05) except for “timely injection” (*p* = 0.13) and “needle dwelling time” (*p* = 0.35). Notably, the proportion reporting “pressing with dry cotton swab” decreased post-intervention (*p* = 0.02), possibly reflecting training emphasis on “no/gentle pressure” recommendations.

##### 3.2.2.3 Comparison of insulin storage assessment before and after QM

Insulin storage knowledge improved significantly post-intervention (*p* < 0.05).

##### 3.2.2.4 Comparison of glycemic control before and after QM

Following the one-year structured QM program, a statistically significant improvement in glycemic control was observed. The mean glycated hemoglobin (HbA1c) level decreased from 7.13% (SD = 1.74%) at baseline to 5.93% (SD = 1.00%) post-intervention. The paired *t*-test confirmed this reduction was significant (*t* = 10.54, *p* < 0.001). The mean reduction in HbA1c was 1.20% [95% confidence interval (CI): 0.98–1.42%]. The effect size (Cohen’s *d* = 0.85) indicates a large intervention effect. A sensitivity analysis excluding participants with extreme HbA1c values (*n* = 5) yielded similar results (mean reduction = 1.18, 95% CI: 0.96–1.40%), confirming the robustness of the findings (Tables 3–6).

**Table 3:** Items with good baseline knowledge/performance in community diabetes insulin injection [*n* = 297 (percentage, %)].

Item	Number (%)
Maintain home insulin stock	287 (96.63)
Spare insulin refrigerated	293 (98.65)
Able to correctly assemble insulin pen independently	285 (95.96)
Know the most suitable body sites for insulin injection	296 (99.66)
Correctly select insulin injection rotation area	291 (97.98)
Clean/disinfect injection area before injection	273 (91.92)
Use alcohol swabs to disinfect injection site	287 (96.63)

**Table 4:** Knowledge assessment before and after special QM [n = 297 (percentage, %)].

Knowledge item	Pre-QM survey result	Post-QM survey result	McNemar χ^2^	*P*
Recognize insulin as a high-alert medication	229 (77.10%)	248 (86.87%)	3.84	0.05
Have received insulin/injection popular science knowledge	154 (51.85%)	295 (99.33%)	181.39	<0.01
Have received standardized insulin operation training	153 (51.52%)	297 (100%)	190.08	<0.01

**Table 5:** Insulin injection operation assessment before and after special QM [*n* = 297 (percentage, %)].

Operation item	Pre-QM survey Result	Post-QM survey Result	McNemar χ^2^	*P*
Complete injection promptly at scheduled time	194 (65.32%)	212 (71.38%)	2.52	0.13
Rotate injection sites	118 (39.73%)	208 (70.03%)	20.69	<0.01
Correct needle dwelling time after injection	178 (59.93%)	190 (63.97%)	1.03	0.35
Press with dry cotton swab after needle removal	261 (87.88%)	239 (80.47%)	6.12	0.02
Single-use and proper handling of needles	41 (13.80%)	268 (90.24%)	347.56	<0.01
Proper disposal of used pen needles	43 (14.48%)	295 (99.33%)	435.94	<0.01
Have undergone re-assessment of injection technique	55 (18.52%)	296 (99.66%)	404.49	<0.01

**Table 6:** Insulin storage assessment before and after special QM [*n* = 297 (percentage, %)].

Storage knowledge item	Pre-QM survey result	Post-QM survey result	McNemar χ^2^	*P*
Know refrigerated insulin needs 30-min warm-up	142 (47.81%)	246 (82.83%)	80.38	<0.01
Know thawed, previously frozen insulin cannot be used	193 (64.98%)	275 (92.59%)	67.73	<0.01
Use opened insulin within stipulated period	222 (74.75%)	269 (90.57%)	25.95	<0.01

## 4 Discussion

The incidence of diabetes in China is progressively rising. National surveys indicate approximately 3,000 new cases daily and 1.2 million annually, presenting significant challenges for community chronic disease management. Achieving glycemic control is crucial to delay progression and complications. This study found a positive correlation between insulin injection use and diabetes duration. As diabetes knowledge dissemination increases, patient resistance to insulin decreases, leading to more patients on insulin therapy. Standardized injection practices are key for glycemic control in these patients (11, 12). The “2014–2015 Global Insulin Injection Technique Questionnaire” revealed widespread non-standard practices globally, with more pronounced issues in China, especially in economically and culturally disadvantaged grassroots communities. Consequences include insulin allergy, lipodystrophy, needle-stick injury, and hypoglycemic coma. As a high-alert medication with a narrow therapeutic index, improper insulin use can more easily induce acute complications and accelerate chronic ones.

The findings of this study are consistent with previous research demonstrating that educational and multidisciplinary interventions can improve insulin injection practices (13, 14). For instance, a study by Bateman et al. (12) reported that optimizing injection technique led to significant reductions in HbA1c, similar to the improvements observed in our cohort. However, unlike hospital-based interventions, our program was implemented entirely in a community setting, highlighting the feasibility and effectiveness of decentralized diabetes care models.

Compared to international data from the 2018 ITQ, our baseline rates of needle reuse (13.8% single-use) and proper disposal (14.48%) were substantially lower than global averages (approximately 30 and 25%, respectively), reflecting the heightened vulnerability of grassroots communities in China (4, 5). Post-intervention, these rates improved to over 90%, surpassing figures reported in some developed countries (15). This suggests that centralized, multidisciplinary management can rapidly close practice gaps, even in resource-limited settings.

The lack of significant improvement in “needle dwelling time” (*p* = 0.35) aligns with findings from other studies indicating that fine motor skills and habitual behaviors are more resistant to change and may require repeated reinforcement and behavioral modeling (16). Future interventions should integrate behavior change theories, such as the Health Belief Model or Social Cognitive Theory, to target deeper cognitive and motivational barriers (17).

With rising diabetes prevalence and the shift of chronic disease management to primary care, developing and implementing targeted strategies for grassroots insulin injection problems can minimize risks, improve diabetes/complication control rates, and reduce primary healthcare burdens. Injection practice problems vary by region, culture, and economy. Community QM for insulin injection should be based on local surveys and targeted strategy research. This study, based on a questionnaire survey in a Zhejiang county community, implemented a special QM program with directed education, training, and evaluation. The ultimate goal was to improve injection standardization, diabetes knowledge, patient compliance, insulin tolerability, and glycemic control. HbA1c is negatively correlated with complications, making glycemic control fundamental for acute and chronic diabetes prevention.

This study demonstrates that a one-year structured special QM program significantly improved insulin injection knowledge, operational standardization, storage knowledge, and reduced HbA1c levels among community patients in the studied county, indicating the effectiveness of localized, targeted interventions. The observed HbA1c reduction of 1.20% is clinically meaningful, as previous research has shown that each 1% reduction in HbA1c is associated with a 21% lower risk of diabetes-related complications (18). The large effect size (Cohen’s *d* = 0.85) further supports the robustness of the intervention’s impact. Compared to 2018 ITQ global data, baseline issues were more severe locally regarding “single-use of needles” (13.8% vs. ~30% global) and “proper needle disposal” (14.48% vs. ~25%), highlighting grassroots community challenges. Post-intervention, standardization rates for needle handling (>90%) approached or surpassed levels reported in some developed countries, suggesting centralized, multidisciplinary management can rapidly close practice gaps. The most significant improvements were in “single-use of needles” and “receiving standardized training,” likely because these behaviors are more amenable to change via direct education, provision of free needles, or enhanced supervision. The lack of significant improvement in “needle dwelling time” (*p* = 0.35) may relate to its dependence on fine motor memory and potentially insufficient training emphasis. Future interventions should incorporate more behavior change theories.

Despite the significant positive outcomes, this study has several limitations that warrant critical consideration. First, the one-group pre-post design, while pragmatic for a community-based quality improvement initiative, lacks a concurrent control group. This design cannot definitively exclude the influence of confounding variables, such as secular trends in diabetes care, seasonal variations in glycemic control, or a potential Hawthorne effect, where patients’ behavior improves simply due to being observed. The significant HbA1c reduction from 7.13 to 5.93% is noteworthy, but its magnitude might be partially attributable to factors other than the intervention itself. Second, the study was conducted in a single county in Zhejiang Province, which may limit the generalizability of our findings to other regions with different socioeconomic statuses, healthcare infrastructures, or cultural practices related to diabetes management. Third, the reliance on self-reported questionnaire data for many outcomes is subject to recall bias and social desirability bias, potentially overestimating the true improvement in practices. The paradoxical decrease in “pressing with dry cotton swab” (*p* = 0.02) likely reflects successful unlearning of an incorrect prior practice, but it also highlights the complexities of self-reported behavioral change. Fourth, the “clinician-nurse-community physician” MCMT model, while effective, was resource-intensive. The economic cost and scalability of this model to other regions with fewer healthcare resources need further evaluation. Finally, the study design did not allow us to disentangle the individual contributions of the four intervention modules (education, training, supervision, feedback) to the overall effect. It is plausible that the combination of strategies was synergistic, but future research using factorial or sequential multiple assignment randomized trial (SMART) designs could help identify the most effective and efficient components.

## 5 Conclusion

This study demonstrates that implementing a structured, multidisciplinary quality management program focused on insulin injection practices significantly improved patient knowledge, operational accuracy, and glycemic control among community-dwelling individuals with diabetes. The program, which included systematic education, hands-on training, and regular supervision, led to marked enhancements in injection technique, storage compliance, and HbA1c levels. These findings underscore the value of targeted, community-based interventions in addressing local gaps in diabetes care. Future efforts should incorporate behavior change theories and expand this model to broader primary care settings to further validate and promote standardized insulin injection practices.

## Data Availability

The original contributions presented in the study are included in the article/supplementary material, further inquiries can be directed to the corresponding author.
