# Supplementary material for: Effectiveness of a structured special quality management program on insulin injection practices among community-dwelling patients with diabetes in a county district: a pre-post intervention study
Source: Front Public Health. 2026 Apr 29;14:1809596. doi: 10.3389/fpubh.2026.1809596 (PMC13168025; doi:10.3389/fpubh.2026.1809596)
Supplement: Supplementary file 1 [file Supplementary_file_1.docx]

**Insulin Injection Practice Questionnaire**

**Section 1: General Information**

1、Your name: ————

2、Gender:
A. Male
B. Female

3、Age:
A. Under 30 years old
B. 31–40 years old
C. 41–50 years old
D. 51–60 years old
E. 61–70 years old
F. 71–80 years old
G. Over 81 years old

4、Education level:
A. Primary school or below
B. Junior high school
C. Senior high school
D. College
E. Bachelor’s degree or above

5、Occupation:
A. Employed
B. Farmer
C. Retired
D. Resigned
E. Other

6、Type of diabetes:
A. Type 1 diabetes
B. Type 2 diabetes
C. Other

7、Duration of diabetes (years):————

8、Duration of insulin therapy (years): ————

**Section 2: Insulin-Related Knowledge**

1. Do you know the type of insulin you are using?
   A. Yes
   B. No
2. Have you ever read educational materials about insulin and insulin injection?
   A. Yes
   B. No
3. Do you consider insulin a high-alert (hazardous) medication?
   A. Yes
   B. No
4. Which of the following are complications related to insulin injection? (Multiple choice)
   A. Lipohypertrophy or lipoatrophy
   B. Infection
   C. Pain
   D. Bleeding and bruising

**Section 3: Insulin Injection Operation**

1. Have you received professional training on insulin injection from medical staff before using insulin?
   A. Yes
   B. No
2. Do medical staff assess your insulin injection technique during regular follow-ups?
   A. Yes
   B. No
3. Do you think regular assessment or guidance on injection technique is necessary?
   A. No need
   B. Needed
4. Do you forget to inject insulin at the scheduled time?
   A. Yes
   B. Occasionally
   C. No
5. Can you correctly assemble an insulin pen independently?
   A. Yes
   B. No
6. Which body sites are suitable for insulin injection? (Multiple choice)
   A. Abdomen
   B. Lateral thigh
   C. Upper outer buttock
   D. Lateral upper arm
7. Do you select the injection area as required?
   A. Yes
   B. No
8. Do you rotate injection sites properly?
   A. Rotate every time
   B. Often rotate (>6 out of 10 injections)
   C. Sometimes rotate (3–6 out of 10 injections)
   D. Seldom rotate (<3 out of 10 injections)
   E. Never rotate
9. Do you clean and disinfect the injection site before every insulin injection?
   A. Yes, every time
   B. Occasionally
   C. Almost never
10. What disinfection method do you use before injection?
    A. No disinfection
    B. Iodophor
    C. Alcohol swab
    D. Water
11. How often do you replace disinfection supplies?
    A. Use until exhausted without checking expiration date
    B. Use within the expiration date
    C. Replace every week
12. How do you pinch the skin during injection?
    A. Pinch up with five fingers together
    B. Pinch with thumb, index, and middle fingers
    C. Pinch with thumb and middle finger
    D. Pinch with thumb and ring finger
13. How long should the needle remain in place after completing insulin injection?
    A. No dwelling time
    B. 2–3 seconds
    C. 6–9 seconds
    D. ≥10 seconds
14. After withdrawing the needle, do you press the injection site? If yes, what do you use? (Multiple choice)
    A. Press with dry cotton swab
    B. Press with alcohol swab
    C. Press with finger
    D. No pressing needed
15. How often do you replace the insulin pen needle?
    A. Single use (one needle per injection)
    B. One needle per day
    C. One needle per week
    D. Replace only after finishing the insulin cartridge
16. Can you correctly dispose of used pen needles? (Correct method: collect and return to medical institutions for sharp waste disposal)
    A. Yes
    B. No
17. Have you or your family members ever been accidentally pricked by a used insulin needle?
    A. Yes
    B. No

**Section 4: Insulin Storage**

1. Do you keep a stock of insulin at home for emergency use?
   A. Yes
   B. No
2. Is your spare insulin stored in a constant‑temperature refrigerator?
   A. Yes
   B. No
3. What is the correct storage temperature for unopened insulin?
   A. Below 0°C
   B. 2–8°C
   C. 10–15°C
   D. 15–30°C
4. How long should insulin be warmed up after being taken out of the refrigerator?
   A. 15 minutes
   B. 30 minutes
   C. 45 minutes
   D. 60 minutes
5. Can frozen insulin be used after thawing?
   A. Yes
   B. No
6. Do you use opened insulin within the valid period specified in the instructions?
   A. Yes
   B. Not noticed
   C. No

Surveyor name: —————
